# Pangenomics in crop improvement—from coding structural variations to finding regulatory variants with pangenome graphs

**DOI:** 10.1002/tpg2.20177

**Published:** 2021-12-13

**Authors:** Silvia F. Zanini, Philipp E. Bayer, Rachel Wells, Rod J. Snowdon, Jacqueline Batley, Rajeev K. Varshney, Henry T. Nguyen, David Edwards, Agnieszka A. Golicz

**Affiliations:** ^1^ Dep. of Plant Breeding, IFZ Research Centre for Biosystems, Land Use and Nutrition Justus Liebig Univ. Giessen Giessen 35392 Germany; ^2^ School of Biological Sciences and Institute of Agriculture Univ. of Western Australia Perth Western Australia Australia; ^3^ Dep. of Crop Genetics John Innes Centre Norwich Research Park Norwich NR47UH UK; ^4^ Center of Excellence in Genomics & Systems Biology International Crops Research Institute for the Semi‐Arid Tropics (ICRISAT) Patancheru India; ^5^ State Agricultural Biotechnology Centre, Centre for Crop Food Innovation, Food Futures Institute Murdoch Univ. Murdoch WA Australia; ^6^ Division of Plant Sciences Univ. of Missouri Columbia MO USA

## Abstract

Since the first reported crop pangenome in 2014, advances in high‐throughput and cost‐effective DNA sequencing technologies facilitated multiple such studies including the pangenomes of oilseed rape (*Brassica napus* L.), soybean [*Glycine max* (L.) Merr.], rice (*Oryza sativa* L.), wheat (*Triticum aestivum* L.), and barley (*Hordeum vulgare* L.). Compared with single‐reference genomes, pangenomes provide a more accurate representation of the genetic variation present in a species. By combining the genomic data of multiple accessions, pangenomes allow for the detection and annotation of complex DNA polymorphisms such as structural variations (SVs), one of the major determinants of genetic diversity within a species. In this review we summarize the current literature on crop pangenomics, focusing on their application to find candidate SVs involved in traits of agronomic interest. We then highlight the potential of pangenomes in the discovery and functional characterization of noncoding regulatory sequences and their variations. We conclude with a summary and outlook on innovative data structures representing the complete content of plant pangenomes including annotations of coding and noncoding elements and outcomes of transcriptomic and epigenomic experiments.

## Core Ideas

- Pangenomes allow integration of many types of DNA variants in a single reference.
- Pangenome studies highlight the importance of structural variations affecting agronomical traits.
- Many structural variations occur in regulatory regions and affect gene expression.
- Adopting pangenome graphs will help understand coding and noncoding variation.

**Abbreviations**
ACR: accessible chromatin region
CNV: copy‐number variation
CNV: copy‐number variation
CRE: cis‐regulatory element
GS: genomic selection
GWAS: genome‐wide association studies
MAS: marker‐assisted selection
PAV: presence‐or‐absence variation
PHG: Practical Haplotype Graph
QTL: quantitative trait loci
SNP: single‐nucleotide polymorphism
SV: structural variation
SV: structural variation or structural variant
TE: transposable element
VG: Variation Graph

## 1 INTRODUCTION

The number of people globally affected by hunger has been rising since 2014 (FAO et al., 2020). Almost 690 million people—8.9% of the world population—are estimated to have been undernourished in 2019. Without change, the world is on track to reach 840 million undernourished people by 2030 (FAO et al., 2020). The reasons for an increasingly undernourished population are manifold including unequal resource distribution, food waste, and crop loss arising from climate change (Barrera & Hertel, 2021; Hasegawa et al., 2016; Janssens et al., 2020). While an integrated approach is vital to successfully curb this alarming trend, climate‐change‐resilient crops are needed to counter more frequent and extreme weather events that affect mostly populations with already high rates of undernourishment (FAO et al., 2020).

During recent decades, significant technological progress has been made in plant breeding to develop new cultivars including marker‐assisted selection (MAS), which has been in use since the early 1990s (Dudley, 1993; Ribaut & Hoisington, 1998). In MAS, genomic markers are identified in silico and used to select individuals for use in specialized breeding programs (Hillel et al., 1990; Tanksley & Nelson, 1996). Originally, trait‐associated genomic markers targeted by these approaches were identified as quantitative trait loci (QTL) (Geldermann, 1975), which in their earliest iteration could contain many genes within the same locus (Beckmann & Soller, 1983; Westman & Kresovich, 1997). The ability to identify candidate genes was accelerated by the construction of reference assemblies representing the DNA sequence of an individual's genome. Reference genomes were used to identify single‐nucleotide polymorphisms (SNPs), copy‐number variations (CNVs), and insertion–deletions (InDels). These markers became the foundation for genome‐wide association studies (GWAS) and genomic selection (GS), where diversity sequencing datasets are compared with reference genomes and the identified variations statistically associated with phenotypes (Crossa et al., 2017; Hayes & Goddard, 2010; Ozaki et al., 2002; Varshney et al., 2005, 2009). The identification of trait‐associated alleles and genes has also been used to drive a revolution in green biotechnology. The first genetically modified crop, the tomato (*Solanum lycopersicum* L.) ‘FlavrSavr’, was released to U.S. markets in the 1990s (Kramer & Redenbaugh, 1994). Since then, commercialization and distribution of genetically modified and, now, gene‐edited crops have the potential to increase yield and improve traits such as biotic and abiotic stresses tolerance (Liu et al., 2021; Shi et al., 2017; Singh et al., 2018; Singh et al., 2020; Varshney et al., 2011; Wang et al., 2014; Zeng et al., 2019).

**Core Ideas**

- Pangenomes allow integration of many types of DNA variants in a single reference.
- Pangenome studies highlight the importance of structural variations affecting agronomical traits.
- Many structural variations occur in regulatory regions and affect gene expression.
- Adopting pangenome graphs will help understand coding and noncoding variation.

The approaches described above require the identification of candidate genes or sequences for modification; however, they usually rely on a single reference genome, which does not contain the full extent of genetic variation present in the species, especially in polyploidy crops (Bayer et al., 2020; Golicz et al., 2016a). This limitation has led to the rise of pangenomes, which combine the genomic data derived from multiple accessions and cultivars to detail the full extent of sequence variation within a species, finding genes and alleles to accelerate crop breeding. The pangenome concept was first proposed by Tettelin et al. (2005) to describe a *Streptococcus agalactiae*, or group B *Streptococcus*, reference combining datasets derived from eight different bacterial isolates (Tettelin et al., 2005). The genes present in all individuals were defined as ‘core’ genes, while the variable fraction was termed ‘dispensable’ (also referred to as ‘accessory’ or ‘variable’). Functional characterization of group B *Streptococcus* core genes highlighted their involvement in essential processes, while a significant portion of disposable genes was found to be the cause of newly acquired traits such as antibiotic resistance (Tettelin et al., 2005). Later pangenomics studies confirmed the critical roles of several variable genes in the organism's adaptation to a certain environment, making them effectively indispensable to the specific strain or cultivar fitness (Scheben et al., 2016). Tettelin et al. (2005) also noted that increasing the sample size would expand the pangenome size indefinitely, making it an ‘open’ pangenome as opposed to a ‘closed’ one, whereby after inclusion of a sufficient number of samples, the addition of further datasets would not result in the identification of novel sequences (Tettelin et al., 2005). Since then, the adoption of high‐throughput and cost‐effective DNA sequencing technologies has resulted in the proliferation of pangenomes, including major crop species such as rice (*Oryza sativa* L.), maize (*Zea mays* L.), soybean [*Glycine max* (L.) Merr.], and rapeseed (*Brassica napus* L.) (Hirsch et al., 2014; Li et al., 2014; Song et al., 2020; Wang et al., 2018; Yao et al., 2015). Compared with single‐reference genomes, pangenomes enable more accurate identification and representation of complex DNA polymorphisms within a species including large insertions, deletions, inversions, duplications, translocations, presence‐or‐absence variations (PAVs,) and copy‐number variations (CNVs). Structural variations (or structural variants, SVs), ranging from few base pairs to several megabase pairs are the result of a variety of mechanisms including transposable elements (TEs) insertion, recombination, and double‐strand breaks repair (Saxena et al., 2014). Structural variations are one of the major determinants of phenotypic variability within a species including many agronomically important traits (Gao et al., 2020; Ledesma‐Ramírez et al., 2019; Song et al., 2020).

Recent studies highlighted the value of analyzing pangenome content beyond the collection of protein coding genes including regulatory regions and repeat content (Gao et al., 2019; Hufford et al., 2021). However, such detailed analysis requires availability of multiple high‐quality genome assemblies and advanced methods for their analysis and visualization, for example using the tools of graphical pangenomics (Eizenga et al., 2020). Pangenomic and comparative analyses are especially challenging for many crop plants that have high repeat content, are ancient, or are recent polyploids.

Here we review recent literature on pangenomics in crops with a focus on their application to find candidate SVs involved in traits of agronomic interest such as disease resistance or flowering time. We highlight the potential of using pangenomes for noncoding regulatory element discovery, functional characterization, and evaluation of SV impact on plant function. We finish with a discussion of innovative data structures representing the complete content of plant pangenomes including the complete repertoire of functional sequences.

## 2 USING PANGENOMES TO DISSECT AGRONOMIC TRAITS

Consistent with early findings that the dispensable genome is enriched with genes involved in environmental responses, pangenomes are being increasingly used in the detection of sequences associated with agronomically relevant traits, such as yield and stress resistance, thereby leading the transition from so‐called genomic‐assisted to pangenomic‐assisted breeding strategies (Table 1).

**TABLE 1:** Agronomical traits addressed by selected pangenome studies highlighting whether a coding or noncoding associated variant was identified

**Species**	**No. of accessions**	**Single reference size**	**Pangenome size**	**Traits studied using the pangenome**	**Variant type**	**Coding or noncoding**	**Reference**
*Brachypodium distachyon* (purple false brome)	54	(Bd21) 272 Mb; 34,310 genes	430 Mb; 37,886 HC genes	Disease resistance, flowering time	PAV	Coding	Gordon et al., 2017
*Brassica oleracea*, *B. macrocarpa* (cultivated and wild cabbage)	9+1	(*Bo* TO1000) 488 Mb; 59,225 genes	587 Mb; 61,379 genes	Disease resistance, flowering time, secondary metabolites	PAV	Coding	Golicz, et al., 2016b
*B. napus* (rapeseed)	53	(Darmor‐bzh v8.1) 850 Mb; 80,382 genes	1,044 Mb; 94,013 genes	Disease resistance	PAV, SNP	Coding	Dolatabadian et al., 2020; Hurgobin et al., 2018
*B. napus* (rapeseed)	8	(Zs11) 960.8 Mb, 100,919 genes	1.8 Gb; 152,185 genes	Silique length, seed weight, flowering time	PAV	Noncoding	Song et al., 2020
*B. rapa* (turnip, napa cabbage)	3	(Chiifu v1.2) 41,019 genes	41,858 genes	Flowering time, stress resistance, lignin formation	PAV	Coding	Lin et al., 2014
*Cajanus cajan* (pigeon pea)	89	(Asha) 606 Mb; 53,612 genes	622 Mb; 55,512 genes	Self‐fertilization, disease resistance, seed weight	SNP, PAV	Coding	Zhao et al., 2020
*Capsicum annuum*, *C. baccatum*, *C. chinense*, *C. frutescens* (pepper)	383	*(Ca* Zunla 1) 35,336 HC genes	4.316 Gb; 51,757 HC genes	Carotenoid and capsaicinoid biosynthetic pathways	PAV	Coding	Ou et al., 2018
*Glycine soja* (wild soybean)	7	(GsojaD, Shandong) 985 Mb; 57,631 genes	986.3 Mb; 59,080 gene families	Disease resistance, flowering time, oil content, height and lodging, yield	CNV, PAV, SNP, InDel	Coding	Li et al., 2014
*G. max*, *G. soja* (cultivated and wild soybean)	29	(Zhonghuang 13) 1.025 Gb; 52,051 genes	57,492 orthologues	Iron uptake	PAV	Coding, noncoding	Liu et al., 2020
*Gossypium hirsutum* (upland cotton)	1581	(TM‐1) 2,347 Mb; 70,199 genes	3,388 Mb; 102,768 genes	Flowering time, morphology, yield, fiber traits	PAV, SNP	Coding, noncoding	Li et al., 2021
*Gossypium. barbadense* (tropical cotton)	226	(3–79) 2,266 Mb; 71,297 genes	2,575 Mb; 80,148 genes	Disease resistance, stress response			
*Helianthus annuus* (sunflower)	493	(HA412‐HO.v.1.1) 3.6 Gb; 52,232 genes	61,205 genes	Disease resistance	SNP, PAV	Coding	Hübner et al., 2019
*Hordeum vulgare* (barley)	20	(Morex V2) 32,878 genes	40,176 orthologues	Yield	PAV inversions	Coding	Jayakodi et al., 2020
*Malus domestica*, *M. sieversii*, *M. sylvestris* (cultivated and wild apple)	91	(*Md* Gala, haploid assembly) 45,352 protein‐coding genes	69,411 orthologues	Fruit quality	SNP, PAV	Coding	Sun et al., 2020
*Medicago truncatula* (barrel medic)	15	(HM101, Mt4.0) 412 Mb; 46,523 genes	431 Mb; 74,700 genes	Pathogen detection	SNP, CNV	Coding	Zhou et al., 2017
*Oryza sativa* (rice)	1483	(Nipponbare) 384 Mb	*indica*: + 52,976 sequences; *japonica*: + 30,349 sequences	Disease resistance, stress resistance, grain width and size	SNP	Coding	Yao et al., 2015
*O. sativa*, *O. rufipogon* (cultivated and wild rice)	66+1	(*Os* Nipponbare) 384 Mb	42,580 genes	Flowering time, stress tolerance, grain weight, tiller angle and plant height, hull color	SNP, PAV	Coding	Zhao et al., 2018
*O. sativa*, *O. glaberrima* (cultivated and wild rice)	3010	(*Os* Nipponbare) 384 Mb	+ 268 Mb; + 12,465 genes	Flowering time, grain length and width, disease resistance	SNP, PAV	Coding	Wang et al., 2018
*Solanum lycopersicum*, *S. cheesmaniae*, *S. galapagense* (cultivated and wild tomato)	725	(*Sl* Heinz 1706, v ITAG3.2) 900 Mb; 35,496 genes	1,179 Mb; 40,369 genes	Disease resistance, fruit flavor	PAV	Coding, noncoding	Gao et al., 2019
*Sorghum bicolor* (sorghum)	176+ 1 (354 for gPAV)	(Moench) 732.2 Mb	883.3 Mb; 35,719 genes	Drought resistance	SNP, PAV	Coding	Ruperao et al., 2021
*Triticum aestivum* (bread wheat)	18+1	(Chinese Spring) 10.7 Gb	∼11 Gb; 140,500 genes	Disease resistance, stress resistance	PAV	Coding	Montenegro et al., 2017
*Zea mays* (maize) pan‐transcriptome	503	(B73) 22,354 RTAs	+ 8,681 RTAs	Flowering time	SNP	Coding	Hirsch et al., 2014
*Zea mays* (maize)	26	(B73) 2,182 Mb	103,538 genes	Disease resistance, flowering time	TE‐insertion; SNP, PAV	Coding, noncoding	Hufford et al., 2021

*Note*. CNV, copy‐number variation; HC, high confidence; InDel, insertion–deletions; PAV, presence or absence variation; RTA, representative transcript assemblies; SNP, single‐nucleotide polymorphism; TE, transposable element.

### 2.1 Disease resistance

The sunflower (*Helianthus annuus* L.) pangenome was recently generated from 493 cultivars, landraces, and compatible wild species to investigate the impact of introgression from wild species on the dispensable genome composition of cultivars (Hübner et al., 2019). Approximately 1.5% of genes were identified as exclusively derived from introgression with wild relatives, including two candidate genes associated with resistance to downy mildew: a syntaxin (*SYP132*), known to contribute to bacterial resistance and PR‐1 protein secretion (Kalde et al., 2007); and a GDSL‐motif lipase involved in plant resistance to fungal infection (Oh et al., 2005). Similarly, multiple pangenomic studies in rapeseed confirmed the role played by dispensable genes in plant defense, with ∼70% of the total predicted resistance genes being found in the variable portion of the pangenome (53 rapeseed accessions) with almost 50% of them absent from the ‘Darmor‐*bzh*’ reference (Hurgobin et al., 2018). Dispensable resistance genes include *BnaA03g43460.1D2*, a potential orthologue of a clubroot resistance gene in *Brassica rapa* L. , *CRa* (Ueno et al., 2012). A later study by Dolatabadian et al. (2020) expanded on this discovery, identifying 753 variable resistance gene analogs (RGA) in the rapeseed pangenome, with 106 resistance gene analog candidates predicted to contribute to blackleg resistance, one of the major diseases affecting *Brassica* species (Dolatabadian et al., 2020; Howlett et al., 2001). Among the dispensable sequences in the *Brachypodium distachyon* (L.) Beauv. pangenome assembly (54 *Brachypodium* lines) (Gordon et al., 2017), *Brdisv1Bd1‐11011965m* was identified as a dispensable gene absent from the reference line (Bd21) and induced during infection of wheat stem rust in Bd1‐1, a line resistant to *Puccinia graminis* f. sp. *tritici* (Figueroa et al., 2013). While the gene encodes for an uncharacterized protein, the region on which this gene is located is syntenic to the stem rust resistance gene *Sr2* locus in wheat (McFadden, 1930), supporting its potential role in *P. graminis* f. sp. *tritici* resistance in *Brachypodium*.

### 2.2 Vernalization and flowering time

As the *Brachypodium distachyon* reference (Bd21) has the shortest flowering time recorded in the species, the pangenome was instrumental in investigating PAVs involved in flowering time variation (Gordon et al., 2017). The gene *Brdisv1ABR21022861m* was absent from both rapid and intermediate flowering lines (including Bd21) but present in all delayed or extremely delayed flowering lines. This gene encodes a NF‐Y subunit transcription factor, a class of transcription factors known to regulate flowering in *Arabidopsis thaliana* (L.) Heynh., wheat, and rice (Kumimoto et al., 2008; Mayer et al., 2014; Wei et al., 2010), confirming the role *Brdisv1ABR21022861m* plays in determining *Brachypodium* flowering time.

In *Brassica napus*, pangenome‐based comparative analysis and PAV GWAS identified variations causing several agronomically relevant traits including silique length, seed weight, and flowering time (Song et al., 2020). Three SVs were found to affect the expression of the same *FLOWERING LOCUS C* (*FLC*) gene, *BnaA10.FLC*, encoding a key transcriptional regulator responsible for delayed flowering and stronger vernalization requirement (Tadege et al., 2001). A LINE (long interspersed nuclear elements) insertion in the first exon of *BnaA10.FLC* detected in spring‐type rapeseed led to low or no vernalization requirement for this ecotype. Conversely, a MITE (miniature inverted‐repeat transposable elements) insertion in the promoter region of *BnaA10.FLC* was found in winter‐type oilseed rape and resulted in a higher expression of *BnaA10.FLC* and stronger vernalization requirement. A *hAT* insertion in the promoter region of *BnaA10.FLC* was identified as the cause for the intermediate vernalization needs of the semi‐winter oilseed rape ecotype, confirming the role played by *BnaA10.FLC* and the impact of SVs in regulating vernalization in rapeseed (Song et al., 2020). In addition to the four *FLC* paralogues identified in the reference sequence for TO1000, *B. oleracea* (L. var. *alboglabra* (L. H. Bailey) Musil] (Okazaki et al., 2007), a *B. oleracea* pangenome assembly led to the identification of another candidate *FLC* gene, *BoFLC2*, which is missing from the reference genome but present in all other lines (Golicz, et al., 2016b). This absence is presumed to be caused by a deletion event that occurred in TO1000 and to be the determinant of the early flowering phenotype of this rapid‐cycling line. Among *B. oleracea* cultivars, curd initiation and flowering time in cauliflower were also shown to be associated with *BoFLC2* in a dose‐dependent manner (Ridge et al., 2015). Finally, a recent maize study combined de novo genome, transcriptome, and methylome analyses of 26 inbred lines to further characterize maize genomic diversity and uncover novel variations in both coding and regulatory regions (Hufford et al., 2021). The resulting pangenome and pan‐epigenome highlighted several SVs and their impact on phenotypes, including TE insertions upstream of transcription start sites of *GL15, ZCN10*, and *Dof21*, three known flowering‐time‐related genes. These insertions were found to correlate with the gene expression levels and, most notably, discern between temperate and tropical lines (Hufford et al., 2021).

### 2.3 Fruit, grain, and seed quality

The pepper (*Capsicum* spp.) pangenome was assembled from 383 cultivars and used as a reference for PAV GWAS analysis to detect deletions in genes involved in carotenoid and capsaicinoid biosynthetic pathways (Ou et al., 2018). A 2.5‐kb deletion in the predicted *Pungent gene 1* (*Pun1*, *pan02g021380*) was uncovered in 50 cultivars with known low capsaicin content, while a deletion in gene *pan06g005570* (a predicted capsanthin–capsorubin synthase) was found in 26 cultivars with yellow or orange fruits. Similarly, 725 tomato accessions were sequenced and compared with the ‘Heinz 1706’ assembly to identify nonreference sequences and build a pangenome comprising both cultivated and wild tomato varieties (Alonge et al., 2020; Gao et al., 2019). Pangenome analysis led to the identification of a shorter, nonreference allele in the *TomLoxC* promoter, which was abundant in wild relatives but negatively selected during domestication, resulting in lower expression levels of *TomLoxC* and a less desirable flavor in cultivated tomatoes. Several modern elite breeding lines originally selected for stress tolerance also showed an unintended and unexpected recovery of this rare allele, with an increase in apocarotenoid production levels in these varieties (Gao et al., 2019). In addition to the examples reported above, a recent soybean pangenome study analyzed nonredundant SVs identified by comparing 29 whole‐genome assemblies to genotype 2,898 accessions. These SVs were used in a pangenome graph‐based PAV GWAS, which lead to the identification of a 10‐kb PAV. The deleted gene located in this region encodes a hydrophobic protein from soybean (HPS) and is associated with seed luster (Liu et al., 2020).

### 2.4 Abiotic stress tolerance

A recently constructed sorghum [*Sorghum bicolor* (L.) Moench] pangenome was used to identify drought‐responsive genes in transcriptomic data generated from both resistant and susceptible genotypes (Abdel‐Ghany et al., 2020; Ruperao et al., 2021; Varoquaux et al., 2019). A total of 79 genes absent from the reference genome were confirmed as differentially expressed during drought stress, including two resistance specific genes, *Sobic.005G069800* and *Sobic.006G127800*, which were found to co‐map with plant height and leaf pigment traits. Analysis of structural variations in a soybean pangenome identified a gene encoding a Fe^2+^/Zn^2+^ regulated transporter associated with iron deficiency chlorosis (Liu et al., 2020).

## 3 PANGENOMES, SVs, AND REGULATORY ELEMENTS—A NEW AVENUE FOR CROP IMPROVEMENT

**FIGURE 1:**
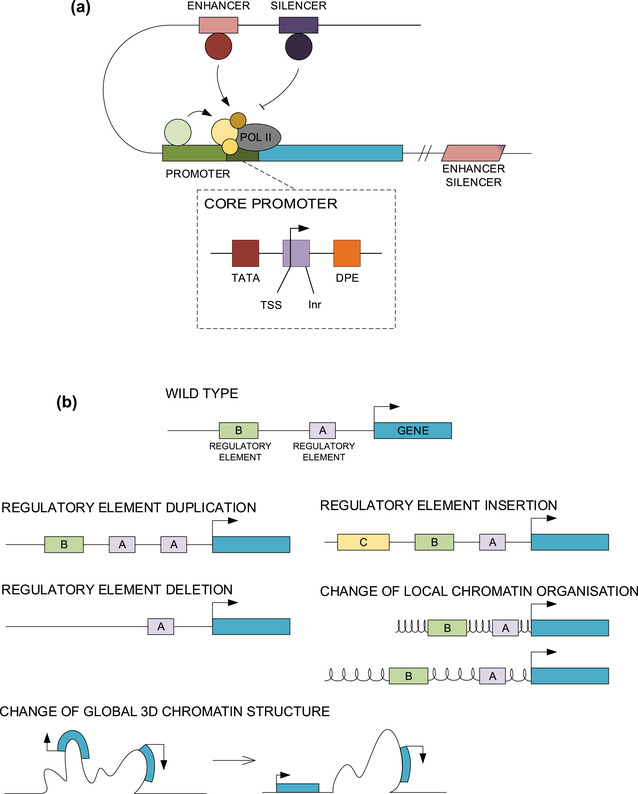
(a) Overview of *cis*‐regulatory elements (CREs) controlling gene expression. The CREs are noncoding DNA sequences capable of recruiting transcription factors and affecting gene expression. The CREs can be broadly subdivided into promoters and enhancers or silencers. Promoters are found directly upstream of the transcription start site, whereas enhancers and silencers can be found megabase pairs (Mbp) away (the promoter configuration shown is not universal). Green box, promoter; pink, enhancer; purple, silencer; blue, gene. (b) Graphical representation of different structural variants potentially affecting gene expression including duplication, deletion, and insertion of entire or segments of regulatory elements, changes in local chromatin organization (or accessibility), and global 3D structure. Purple, green, yellow boxes (A, B, C), regulatory elements; blue, gene

As outlined above, while variations in coding regions have been the major focus of functional studies thus far, recent reports highlight the importance of SVs affecting *cis*‐regulatory elements (CREs) including promoters and enhancers (Figure 1a). Structural variations can potentially affect gene expression via several mechanisms including regulatory element insertion, duplication, deletion, and disruption of the three‐dimensional chromatin structure (Figure 1b) (Chiang et al., 2017; Doğan & Liu, 2018). From a plant breeding perspective, regulatory variants are especially valuable because of their potential to provide a spectrum of trait variation by fine tuning gene expression and precise manipulation of quantitative traits (Rodríguez‐Leal et al., 2017). Changes in CREs, which are often modular and tissue‐specific, are also predicted to be less pleiotropic than those in protein‐coding genes (Wittkopp & Kalay, 2011). Alterations of the regulatory regions are therefore expected to result in more subtle phenotypic effects.

The introduction of chromatin accessibility assays, such as ATAC‐seq (Buenrostro et al., 2013), allowed for genome‐wide identification of accessible chromatin regions (ACRs), which often coincide with regulatory elements. This technique was originally developed for animal and human research, though several protocols have now been optimized for plant tissues and applied to study ACR distribution in major crops such as maize, wheat, and barley (*Hordeum vulgare* L.) (Concia et al., 2020; Lu et al., 2019; Ricci et al., 2019; Sun et al., 2020). In combination with ACR profiling, the application of long‐read sequencing and pangenomics provides a powerful approach for the assessment of the effects of structural variants on CREs and gene expression.

### 3.1 Effect of SVs on gene expression

A recent study on tomato reported that 95% of the 34,075 annotated genes had at least one SV within 5 kb of the coding sequence across the 100 genomes analyzed (Alonge et al., 2020). Approximately 10% of variants occurring in regulatory regions were associated with changes in expression vs. ∼50% of variants affecting coding regions. Consistent with predicted subtler effects of regulatory variants, SVs affecting regulatory regions were reported to have less impact on gene expression than those affecting coding sequences, with mean log_2_ fold change of 1.36 and 2.47, respectively (Alonge et al., 2020). In maize, expression QTL (eQTL) analysis using kernel transcriptome data from 368 maize inbred lines confirmed that while eQTLs with a greater effect tended to overlap genes, the majority (∼80%) of expression‐associated SVs were found in intergenic regions (Yang et al., 2019). Furthermore, by proportion, eQTLs were seven times more likely to be detected using SVs rather than SNP markers, suggesting that SVs have stronger impact on gene expression than SNPs.

The study of regulatory regions will be of particular interest with regard to pangenomes of polyploid crops, where multiple copies of genes exist and alterations in CREs can result in neofunctionalization by promoting changes in timing and location of gene expression. A study in rapeseed, for example, showed that flowering time genes are preferentially retained post‐polyploidization, resulting in diverged expression patterns between homologues, most likely resulting from CRE divergence (Jones et al., 2018).

### 3.2 TEs as novel regulatory elements

Transposable elements are a known driver of SVs, and they have been shown to shape the regulatory landscape of crop plant genomes either by the disruption of existing or the generation of new regulatory elements (Chuong et al., 2017; Feschotte, 2008; Gill et al., 2021). In maize, the comparison of four lines identified almost 3,000 ACRs contained within TEs and several hundred that overlapped a TE insertion in at least one of the lines. The TE insertions were shown to be associated with changes in methylation, chromatin accessibility, and, potentially, regulatory functions. TEs carrying ACRs were enriched for association with higher expression of nearby genes, suggesting they contribute novel regulatory elements (Noshay et al., 2021). Beyond genome‐wide analyses, individual examples also highlight the role of TEs in gene expression regulation. A TE (*Hopscotch*) inserted in a regulatory element of maize *teosinte brached1* (*tb1*) was shown to act as an enhancer and partially explained apical dominance in maize (Studer et al., 2011). Also in maize, *Ac*/*fAc*, a *hAT* family element that can undergo transposition using termini of two adjacent elements, was shown to induce expression of *pericarp color 2* gene (*p2*) by capturing the enhancer sequence of another gene (Su et al., 2020). In *Brassica napus*, a CACTA‐like TE inserted upstream of a P450 monooxygenase (*BnaA9*.*CYP78A9*) led to an increase in silique length and seed weight (Shi et al., 2019). In addition, insertion of a highly methylated COPIA‐like long terminal repeat retrotransposon in the promoter region of *SHATTERPROOF1* homologue resulted in the repression of its expression and shattering resistance in rapeseed (Liu et al., 2020). Considering the key role played by TEs in generating SVs and modulating plant yield and fitness, their precise identification and annotation are among the outstanding challenges to generating high‐quality pangenomes (Coletta et al., 2021).

### 3.3 Editing regulatory elements for crop improvement

Mounting evidence supports the importance of regulatory region variation for crop improvement. In maize, a strong relationship was observed between the genetic variation in putative regulatory elements and complex trait variation (Rodgers‐Melnick et al., 2016). The divergence of *cis*‐regulatory regions associated with domestication also underscores their important roles in the control of traits targeted by artificial selection (Lemmon et al., 2014; Wang et al., 2017). Considering the lower potential for pleiotropic effects, CREs constitute attractive genome editing targets.

**FIGURE 2:**
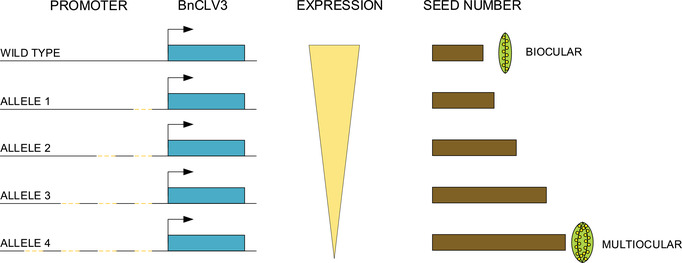
Genome editing of *cis*‐regulatory elements: a hypothetical scenario of editing of *Brassica napus CLV3* homologues’ *cis*‐regulatory elements to generate multiocular siliques and range of variation in seed number. *Brassica napus* has two, mostly redundant, copies of *BnCLV3*, so editing of both would be necessary (Xu et al., 2021; Yang et al., 2018)

In tomato, inspired by the natural variation observed between wild and domesticated relatives, researchers used CRISPR‐Cas9 mutagenesis to generate novel alleles of the promoter of *SlCLV3*, a gene affecting fruit size and engineered a continuum of phenotypic variation. It was noted that although patterns were observed, with larger deletions having the most impact, the magnitude of the phenotypic effect could not be easily predicted from mutations alone, suggesting a complex interplay between CREs controlling gene expression (Rodríguez‐Leal et al., 2017). Similarly, in maize, it was possible to engineer a spectrum of variation for yield‐related traits by targeting homologues of *CLV3* (Liu et al., 2021). It is conceivable that facilitated by pangenomics‐based CRE identification and functional characterization, similar approaches can be applied to other species and candidate genes (Figure 2).

## 4 PANGENOME GRAPH—UNIFYING FRAMEWORK FOR PANGENOME ANALYSIS

**FIGURE 3:**
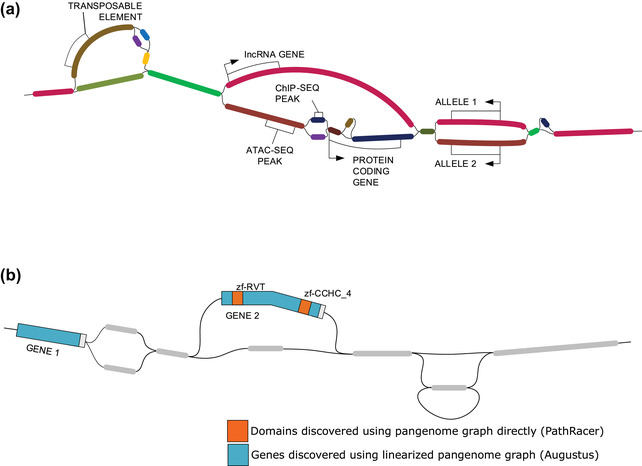
Pangenome graph annotation. (a) A hypothetical example of pangenome graph annotation and visualization integrating multiple layers of information including annotation with coding and noncoding loci, regulatory elements, results of transcriptomic, and ChIP‐seq and ATAC‐seq experiments. Annotating pangenome graph directly allows discovery of features spanning multiple segments (nodes). (b) Actual functional annotation of a segment of pangenome graph representing *Brassica napus* chromosome A01. The graph was built using minigraph with Darmor‐bzh (Rousseau‐Gueutin et al., 2020) and Express 617 (Lee et al., 2020) assemblies and annotated using PathRacer (applied directly to the pangenome graph (only selected domains were used in the search); Shlemov & Korobeynikov, 2019) and Augustus (applied to a linearized version of the pangenome graph; Stanke et al., 2006; linearized pangenome was obtained with gfatools gfa2fa)

Construction of high‐quality reference genomes of multiple individuals of the same species has become the norm. The availability of corresponding epigenomic and transcriptomic data has enabled the functional annotation of these references—from coding and noncoding genes to regulatory elements. The challenge is the accurate representation of the wealth of available information (Jayakodi et al., 2021). Several pangenomic models have been established thus far—from simple collections of unaligned sequences to graphical representations. Pangenome graphs can be used to represent the sequence content and the corresponding functional annotation of an entire population, species, or a clade by compressing redundant sequences into smaller data structures while retaining information on genomic diversity and whole‐genome relationships (Eizenga et al., 2020) (Figure 3). Graphs are composed of the DNA sequences (nodes), links between them (edges), and information about arrangement of nodes found in each constituent genome (paths). Paths provide a stable coordinate system allowing management of positions, annotations, and alignments across multiple genomes and transitions between graph and linear coordinates. To date, several approaches for pangenome graph construction have been adopted including saturation of the reference sequence with variants and using whole genome alignments. The Variation Graph (VG) toolkit (Garrison et al., 2018) can be employed to transform a reference sequence and variation file in Variant Call Format (VCF) into a pangenome graph and build pangenome from multiple genome assemblies (pggb from VG developers). It also contains a suite of tools for mapping of reads to the pangenome graph, variant genotyping, and projection of linear annotations. Minigraph can be used to generate graphs from assembled genomes, map sequences to graphs, and call structural variants (Li et al., 2020).

The main obstacle to the widespread adoption of pangenome graphs is the lack of suitable bioinformatics tools, as those designed for linear genomes are not readily transferable. These include tools for read mapping, small and large variant calling and genotyping, haplotype inference, gene annotation, homology searches, epigenomic, transcriptomic, association studies, and pangenome visualization (Table 2). Integrative genomics approaches have been shown to be invaluable for sequence functional annotation (Golicz et al., 2018; Hassani‐Pak et al., 2021). Ideally, a complete and fully annotated pangenome graph would integrate genomic, epigenomic, and transcriptomic datasets, thus facilitating downstream functional and comparative analyses (Figure 3).

**TABLE 2:** Overview of key bioinformatics analytical tasks and corresponding tools that can be used for liner genome and pangenome graph analysis

Application	Genome tool	Pangenome tool	Notes
Read mapping	Bowtie2 (Langmead & Salzberg, 2012), BWA (Li & Durbin, 2009), BWA MEM (Li, 2013), HISAT2 (Kim et al., 2019)	VG map (Garrison et al., 2018), GraphAligner (Rautiainen & Marschall, 2020), Minigraph (H. Li et al., 2020), Giraffe (Sirén et al., 2020)	VG map and Minigraph map both short and long reads. GraphAligner maps long reads only. Giraffe maps only short reads. HISAT2 is spliced read alignment (RNA‐seq) enabled and can also account for variants by building acyclic variation graph.
Single‐nucleotide variant (SNV) genotyping	GATK (Poplin et al., 2018), FreeBayes (Garrison & Marth, 2012), Bcftools (Li, 2011)	Graphtyper (Eggertsson et al., 2017)	Many linear genome tools perform both SNV discovery and genotyping.
Structural variation (SV) genotyping	SVTyper (Chiang et al., 2015), Nebula (Khorsand & Hormozdiari, 2021), SVJedi (Lecompte et al., 2020), Sniffles (Sedlazeck et al., 2018)	Paragraph (Chen et al., 2019), GraphTyper2 (Eggertsson et al., 2019), VG toolkit (Garrison et al., 2018)	Minigraph can also be used for SV calling.
Gene annotation	MAKER2 (Holt & Yandell, 2011), BRAKER2 (Brůna et al., 2021), FINDER (Banerjee et al., 2021), Funannotate (Palmer & Stajich, 2017)	Not available	Performing independent annotation of individual genomes may lead to artefacts (Bayer et al., 2017; König et al., 2016) Annotations of linear genomes can be projected onto graph using VG annotate/rna.
Homology searches	BLAST (Camacho et al., 2009), DIAMOND (Buchfink et al., 2015), HMMER (hmmer.org)	PLAST (Schulz et al., 2021), PathRacer (Shlemov & Korobeynikov, 2019)	PLAST is pangenome‐graph counterpart to BLAST (currently only supports graphs built by Bifrost). PathRacer aligns profile HMM directly to the assembly graph (an experimental version supporting minigraph gfa files is available).
ChIP‐seq peak calling	MACS (Zhang et al., 2008)	Graph Peak Caller (Grytten et al., 2019)	Graph Peak Caller is based on MACS and VG.
Transcript mapping and quantification	HISAT2 (Kim et al., 2019), STAR (Dobin et al., 2013), Kallisto (Bray et al., 2016), featureCounts (Liao et al., 2014), RSEM (Li & Dewey, 2011)	VG rna (constructs spliced graph), VG mpmap (maps reads to spliced graph), RPVG (quantifies transcript expression)	Pangenome graph‐based workflow improves accuracy over state‐of‐the‐art RNA‐seq mapping methods (Sibbesen et al., 2021). It allows quantification of haplotype‐specific transcript expression.
Association studies	Plink, TASSEL (Bradbury et al., 2007), GAPIT (Lipka et al., 2012), GenABEL (Aulchenko et al., 2007)	Pangenome‐wide association studies (approach is based on frequented regions in pangenome graph; Manuweera et al., 2019)	Structural variations genotyped from a pangenome graph can be projected onto a linear reference and the results can be used for analysis using standard linear genome tools, adjusting for population genomics assumptions.
Visualization	GBrowse (Stein, 2013), JBrowse (Buels et al., 2016), Tablet (Milne et al., 2010), IGV (Robinson et al., 2011), MUMmer (Marçais et al., 2018), SyMAP (Soderlund et al., 2006)	Bandage (Wick et al., 2015), MoMI‐G (Yokoyama et al., 2019), Sequence Tube Maps (Beyer et al., 2019), GfaViz (Gonnella et al, 2019), Pantograph (https://graphgenome.org/)	One of the standing challenges of pangenome visualization tools is display of metadata and annotations.

*Note*. For linear genomes, the most popular or representative tools are shown although many more exist.

Despite the early stage of pangenome graph‐specific tool development, several practical applications have already emerged. Practical Haplotype Graph (PHG) is a graph‐based computation framework and associated pipeline for inference of high‐density genotype from low coverage (skim) sequencing. A PHG was employed in maize to impute genotypes of recombinant inbred lines of a NAM population with an average accuracy of over 99%. Compared with standard genotype files, PHG increased the efficiency of data storage by four orders of magnitude (30,000‐fold) (Franco et al., 2020). Graph Peak Caller is another tool designed specifically for the identification of ChIP‐seq peaks using pangenome graph as a reference. Analysis of *Arabidopsis* 1000‐genomes data showed that the combination of a VG‐constructed graph and Graph Peak Caller identified peaks overlapping sequence not found in the linear reference. The peaks found were generally more motif enriched, suggesting higher‐quality calls (Grytten et al., 2019). Recently a VG‐based pantranscriptome pipeline became available, which allows construction of spliced pangenome graphs, mapping of RNA sequencing data, and haplotype‐aware expression quantification (Sibbesen et al., 2021). The pangenome graph also provides a convenient framework for the genotyping of SVs across a large number of individuals, for example, for use in SV GWAS studies (Liu et al., 2020; Ruperao et al., 2021; Song et al., 2020; Zhao et al., 2020). The availability of comprehensive, well‐annotated pangenome graphs including both genes and regulatory elements will become a key stepping‐stone for the next generation of genomic analyses.

## 5 CONCLUSIONS

While most pangenomics studies to date have focused on SVs affecting coding regions, the importance of their impact on the regulatory elements, including promoters and enhancers, has become apparent. Availability of genome‐wide chromatin accessibility profiling technologies, which can be applied to multiple species and tissue types, allows inclusion of CRE profiling in pangenome analyses. This will enable, for example, the assessment of CRE diversity within species, evaluation of the SV impact on gene expression, and estimation of the relative contributions of CRE and coding sequences diversity to the phenotypic differences observed. Inclusion of crop‐wild relatives in pangenomes, referred as ‘super pangenomes’ (Khan et al., 2020), will improve the identification of CREs targeted by artificial selection (Lemmon et al., 2014; Wang et al., 2017), providing new genome editing targets. Simultaneously, expansion of pangenome studies to higher taxonomic units will help identify core conserved regulatory modules and species‐specific layers of regulation. Widespread adoption of the pangenome graph as a reference will allow for greater integration and varying data types, facilitating functional and comparative analyses to develop the next generation of climate change resilient and high‐performance crops.

## AUTHOR CONTRIBUTIONS

Silvia F. Zanini: Investigation, Methodology, Resources, Visualization, Writing – original draft, Writing – review & editing. Philipp E. Bayer: Investigation, Methodology, Resources, Writing – original draft. Rachel Wells: Conceptualization, Writing – review & editing. Rod J. Snowdon: Conceptualization, Writing – review & editing. Jacqueline Batley: Conceptualization, Writing – review & editing. Rajeev K. Varshney: Conceptualization, Writing – review & editing. Henry T. Nguyen: Conceptualization, Writing – review & editing. David Edwards: Conceptualization, Writing – review & editing. Agnieszka A. Golicz: Conceptualization, Resources, Visualization, Supervision, Funding acquisition, Writing – original draft, Writing – review & editing.

## CONFLICT OF INTEREST

The authors declare no conflicts of interest.
